# Measurement of Acceleration Response Functions with Scalable Low-Cost Devices. An Application to the Experimental Modal Analysis

**DOI:** 10.3390/s21196637

**Published:** 2021-10-06

**Authors:** Alvaro Magdaleno, Juan J. Villacorta, Lara del-Val, Alberto Izquierdo, Antolin Lorenzana

**Affiliations:** 1 School of Industrial Engineering, ITAP, University of Valladolid, Paseo del Cauce 59, 47011 Valladolid, Spain; alvaro.magdaleno@uva.es (A.M.); lvalpue@eii.uva.es (L.d.-V.); 2 Department of Signal Theory and Communications and Telematic Engineering, University of Valladolid, Paseo de Belén 15, 47011 Valladolid, Spain; juavil@tel.uva.es (J.J.V.); alberto.izquierdo@tel.uva.es (A.I.)

**Keywords:** scalable low-cost SHM system, MEMS accelerometers, myRIO platform, non-destructive testing, experimental modal analysis

## Abstract

One of the most popular options in the Structural Health Monitoring field is the tracking of the modal parameters, which are estimated through the frequency response functions of the structure, usually in the form of accelerances, which are computed as the ratio between the measured accelerations and the applied forces. This requires the use of devices capable of synchronously recording accelerations at several points of the structure at high sampling rates and the subsequent computational analysis using the recorded data. To this end, this work presents and validates a new scalable acquisition system based on multiple myRIO devices and digital MEMS (Micro-Electro-Mechanical System) accelerometers, intended for modal analysis of large structures. A simple form of this system was presented by the authors in a previous work, showing that a single board with some accelerometers connected to it got to obtain high quality measurements in both time and frequency domains. Now, a larger system composed by several slave boards connected and synchronized to a master one is presented. Delays lower than 100 ns are found between the synchronised channels of the proposed system. For validation purposes, a case study is presented where the devices are deployed on a timber platform to estimate its modal properties, which are compared with the ones provided by a commercial system, based on analog accelerometers, to show that similar results are obtained at a significantly lower cost.

## 1. Introduction

The need for cost-effective systems capable of detecting and recording the dynamical response of large and relevant structures is undeniable. During the last decades, the concern about the preservation of structures and infrastructures has been constantly increasing, but the high number of historical buildings and civil structures that need to be monitored makes it a complicated and barely affordable problem. To help assess the state of potentially damaged buildings, technicians use a variety of procedures. Among them, the non-destructive techniques (NDT) and those which are little or non-invasive for the structure under study are the most interesting ones. In this sense, the material that constitutes the structure should ideally suffer no extra damage during the health assessment process, and at the same time, provide a precise idea of the state of the structure to decide the type of maintenance work that need to be carried out. The application of such techniques can be applied to any type of structure to help the companies and governments to foresee maintenance works and extend their lifespan, which in the long term, implies important economical savings and an engagement with the environmental care.

Among the different types of techniques that meet the critical aspects mentioned above, those based on monitoring the dynamical response of the structure have attracted much interest during the last few years [1] for being robust and harmless for the structure under test. With a set of well-placed sensors, a series of time signals can be collected from the structure and processed to estimate its modal or physical properties. The evolution of these properties over time can be helpful to assess the actual state of a structure and locate the potential damage it may suffer [2,3]. In addition, monitoring the ambient properties like temperature or humidity can be of great importance in order to correlate the estimated properties with them and separate the deviations due to atmospheric phenomena from true damage. However, for these techniques to be truly effective, the sensors need to be permanently installed on the structure, continuously recording the structural response, transferring the recorded data to a remote server and providing trustfully information about its current state [1,4,5,6]. Unfortunately, currently, this is a hard challenge that commercial structural health monitoring (SHM) systems are not fully prepared to undertake. There are many monitoring systems intended to identify the structural properties, some permit to incorporate ambient properties measurement, but few are conceived to work continuously. Finally, none of the commercially available SHM systems satisfy another important restraint: its cost. As mentioned above, the SHM system which can help to maintain historical buildings and civil structures is intended to be installed on a high number of structures, and this is a field for which the investment is usually low. For that reason, and in order to be truly attractive for actual use, the monitoring system needs to be as affordable as possible.

Among the variety of sensors that can be used to perform a continuous monitoring of the dynamical structural response, accelerometers are the most spread ones due to their versatility, durability and reduced cost [7]. This type of sensor is intended to measure the acceleration response of a certain point of the structure in one, two or three directions and, as mentioned before, are helpful to estimate the modal properties of the structure (namely, its natural frequencies, its damping ratios and its mode shapes) [8]. In the literature, a variety of examples of SHM systems based on acceleration measurements of industrial machinery, wind turbines or civil structures [9,10,11,12] can be found. However, for civil building structures, these techniques have only been applied as early warning systems (EWS) in areas with severe seismic activity [13], and less intensely as an NDT on historic timber structures [14]. There exist a variety of types of accelerometers, being the ones based on piezoelectric crystals and those embedded in Micro Electro-Mechanical Systems (MEMS) the most spread ones [15,16,17]. In general terms, the piezoelectric sensors produce higher quality measurements than the MEMS sensors, but at a significant higher cost. The authors presented in a previous work [18] a low-cost system based on MEMS accelerometers that is capable of acquiring high quality measurements at a fraction of the cost of a commercial system based on piezoelectric sensors.

Certain specific SHM applications require the use of other types of sensors. For example, strain gauges are widely used to monitor strain and stress variations [19,20]. Displacements can be measured by means of Linear Variable Differential Transformers (LVDTs) [21], or in certain situations, a combination of accelerometers and a Global Position System (GPS) [22,23]. Finally, sensor hybridization techniques are also interesting to detect potential sensor damage or failure [20].

To be considered as such, an SHM system not only requires a set of sensors but also a proficient data logger. In this sense, the low-cost system presented in the previous paper [18] was said to be scalable and potentially useful to estimate the structural modal properties, but only one device was used to monitor a simple timber beam and estimate its frequency response functions (FRFs). When scaling the system, several devices need to be connected to deal with larger amounts of sensors. In this process, a number of issues may arise in terms of signal synchronization, i.e., measuring all the data points from all the sensors as simultaneous as possible (in other words, with the least amount of time shift between them). A deficient synchronization between the different devices may lead to appreciable phase shifts in the estimated FRFs, which leads, in turn, to severe deficiencies in the extracted modal properties required to perform the maintenance works prediction. Certain works [24,25,26,27], are devoted to presenting synchronized sensor sets for structural health monitoring. The authors show in [24] a way to synchronize two wireless accelerometers, but the procedure is not applied to larger sensor networks and the recorded data is not further used to estimate any structural property. The authors in [25] perform a complete modal analysis with the recorded signals of a wireless network of synchronized sensors, but they do not quantify the time shift that exist between simultaneous samples (i.e., associated to the same timestamp) in their system. A synchronized acquisition system is developed in [26] and applied to perform a modal analysis of a simple beam by means of five sensors, but they do not provide an analysis of the actual time shifts between simultaneous data. Finally, the authors in [27] present a distributed architecture based on analog accelerometers which are synchronized by using multiplexing techniques, which is an alternative approach to the one proposed here.

In this work, the low-cost system is further developed with respect to the previous work of the same authors and three devices, similar to the one presented there, are connected and synchronized to perform a set of tests devoted to quantifying the maximum delay that exist in the measured signals. Although the synchronization technique is similar to the one presented in [27], the use of digital MEMS sensors directly permits to reduce the frequency synchronization clock, which enables to use longer cables and thus distribute them over longer distances. The three synchronized devices are then deployed on a medium-sized structure to simultaneously record the response of a total of 12 MEMS accelerometers. To show the goodness of the recordings and the device synchronization, a complete experimental modal analysis procedure, which is a demanding analysis that requires all the registered data to be properly synchronised to provide meaningful results, is carried out with software programmed in the same LabVIEW environment that helps to control the SHM system. The estimated modal properties, including the mode shapes, which may be influenced by acquisition errors, are compared to the ones provided by the commercial system used as a reference. This reference system, presented in the previous work [18], is based on piezoelectric accelerometers and the SIRIUS^®^ datalogger from Dewesoft^®^ [28], specifically designed for this type of analysis. As a case study, the proposed system is applied to a timber structure. This type of structure has certain characteristics and can be monitored by means of different technologies [29]. Certain make use of low-cost systems [30], different to the one proposed in this work. Finally, the main contribution of this work to the Sensors Science is the use of digital MEMS accelerometers, which are integrated in a low-cost distributed system. This system is based on devices which have their own FPGA that can be efficiently synchronised.

The manuscript is organized as follows. Section 2 is devoted to summarizing the low-cost device and to describing the synchronization aspects. Section 3 presents the structure, the sensor set-up, the recording configuration and the subsequent analysis details. Finally, Section 4 summarizes the main results, with a thorough discussion about them, and Section 5 presents the main conclusions and remarks.

## 2. Monitoring System Description

### 2.1. System Architecture

**Figure 1:**
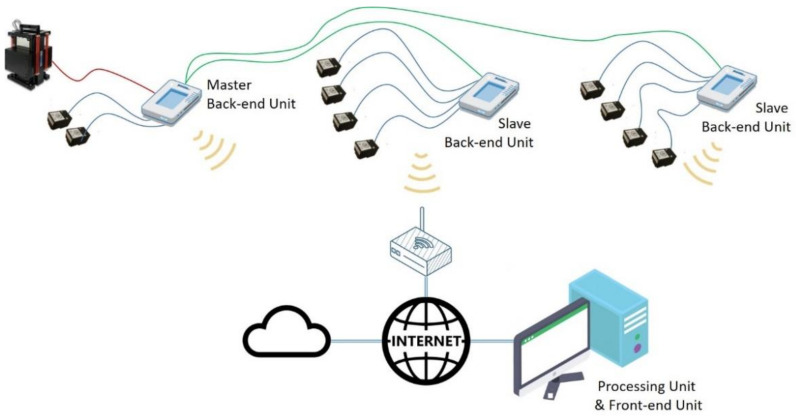
Configuration of the monitoring system.

In order to attain the pursued objectives, the system must be scalable, since it allows varying the number of accelerometers to be used; configurable; thus, such that the position of the sensors can be adapted to the structure to be monitored; and spatially distributed, as schematically shown in Figure 1. It is based on the wireless interconnection of the Back-end units with both the Front-end unit, devoted to acquiring data from the sensors, and the Processing unit, devoted to handling the registered information. In this way, it is possible to configure a global system with a large number of sensors. The different units that may be present in the system are synchronized with each other, with one of them acting as the control unit (Master). For this reason, the proposed set-up comprises a total of 3 units, the minimum number required to measure the longest delay (that occurs between two Slave Back-end units).

**Figure 2:**
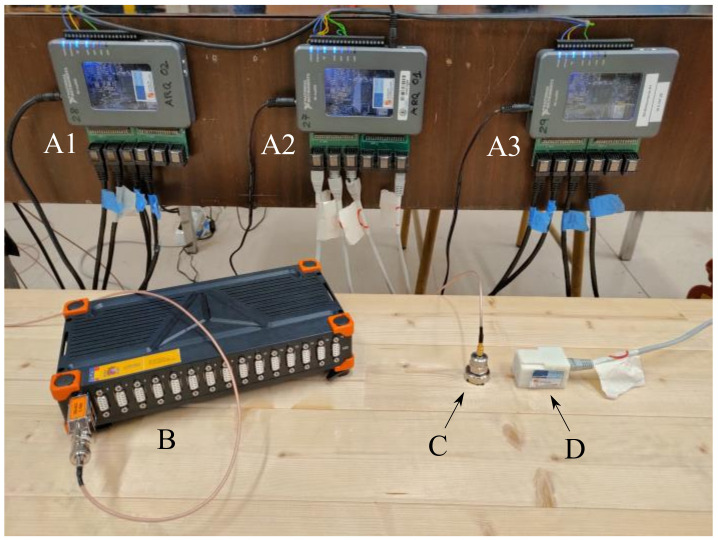
Three synchronized Back-end units (**A1**–**A3**), the commercial acquisition system (**B**) and two sensors: one piezoelectric accelerometer (**C**) and a digital MEMS accelerometer (**D**).

The acquisition system is based on a set of ADXL355 digital MEMS accelerometers from Analog Devices [31], which, distributed in groups, are configured and managed by Adaptation units, or Back-end units, based on a myRIO device from National Instruments [32], as shown in Figure 2, letters A and D. These Back-end units gather the data provided by the sensors and transfer it to the Processing unit. The Back-end units can also control the actuators, such as inertial devices or shakers, by generating and transferring the corresponding acting signals to them. These devices are useful to induce vibration on a structure in a controlled way. By correlating the measured acting force with the measured response, the FRFs of the structure can be computed as shown in [18] and later in this work.

The Processing unit, implemented in this case in a PC, oversees the whole acquisition procedure and processes the registered signals in both the time and the frequency domains. This unit is also responsible for storing the registered data and the computed results in a cloud database, and for carrying out the modal analysis procedure of the structure under study.

The measurements and the calculation process are supervised by a Control unit, or Front-end unit, that it is also implemented on a PC in this case. This Front-end unit is in charge of the interaction with the user, as it helps to send commands to the whole system and visualize both the recorded data and the computed results. It also manages all the Back-end units connected to the system using a Wi-Fi interface and is responsible for configuring the accelerometers connected to each myRIO device, controlling the start and the end of the acquisition, as well as any problem that may arise during the whole process.

### 2.2. System Synchronization

To achieve a synchronous acquisition in distributed systems, it is necessary that all the involved units share the same sampling clock and a start signal or trigger [27,33]. To obtain these common elements, there are two main synchronization approaches: signal-based methods and clock-based methods. The signal-based synchronization approach requires sharing the clock and trigger signals directly among all the involved units. Conversely, in clock-based synchronization methods each unit obtains the clock signal from an external time reference using network protocols such as the Time Sensitive Network (TSN) or the IEEE 1588.

The choice of the most suitable synchronization method depends on parameters such as the number of units to be synchronized, the distance between them, the clock frequency or the required accuracy. In the proposed system, the number of units will be moderate, the distance between them will be less than 100 m, with an acquisition frequency of 4 kHz and with high accuracy; thus, a signal-based synchronization has been chosen.

The system synchronization is carried out between the Back-end units. One of the units, acting as the Master, is in charge of generating the synchronization sampling clock and the trigger signal that is sent to the rest of the Back-end units, which act as Slaves, through point-to-point cables. Each Slave Back-end unit reads the trigger signal inside its FPGA with a frequency of 160 MHz; thus, such that the delay at the beginning of the acquisition is less than 6.25 ns. In the same way, the synchronization clock signal is checked with the same frequency of 160 MHz, reconstructing the sampling clock in each Slave Back-end unit and sending it to the MEMS accelerometers to set the sampling times. In this way, clock drift due to differences in the internal oscillators of each unit is avoided and, at the same time, jitter is reduced by ensuring that all the accelerometers in the system acquire the data with sufficient synchrony.

## 3. Validation of the Proposed Distributed System: Synchronization

### 3.1. Reference System

In order to compare the performance of the proposed system, a commercial one based on analog piezoelectric accelerometers, specifically conceived for high performance data acquisition and modal analysis, is used as the reference system. The accelerometers, which are of the brand MMF and have a nominal sensitivity of 100 mV/g [34], are connected to a high-end datalogger, a SIRIUS device of the brand Dewesoft [28]. Both are shown in Figure 2B,C. Up to 16 accelerometers (or other types of sensors) can be simultaneously connected to the datalogger to record up to 200 kS/s through each 24-bit analog to digital converter. The main characteristics of this system are summed up in Table 1 together with the same characteristics of the proposed low-cost system for comparison purposes [18].

### 3.2. Synchronization Tests

The synchronization between the Back-end units has been assessed to verify that the acquisition is properly performed. Thus, three myRIO devices were used, the same number that are used in subsequent test. One of them acts as Master Back-end unit and the other two are the Slaves Back-end units, each one connected by a 1 m long synchronization cable to the Master unit. The sampling clock reconstructed by each myRIO was compared with the global synchronization clock, using a 4-channel digital oscilloscope.

**Figure 3:**
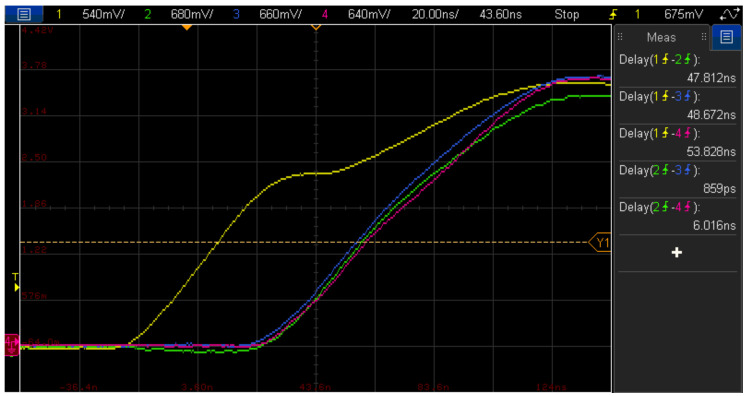
Global sync clock (yellow) and sampling clocks of Master Back-end units (green) and two Slave Back-end units (blue and red).

Figure 3 shows a screenshot of the oscilloscope screen showing the overall sync signal (yellow) generated by the Master unit and the acquisition clocks of both the Master unit (green) and the two Slave units (blue and red). There is a delay between the synchronization clock and the Back-end acquisition clocks of about 50 ns, which is partly due to the propagation delay through the synchronization cable, although it also includes a fixed delay due to the clock regeneration algorithm. Conversely, it is observed that all the sampling clocks, reconstructed by each Back-end unit, are almost in phase, with a delay between them below 10 ns.

Using the advanced features of the oscilloscope, the relative delays between the clocks were measured and the results obtained after 10,000 experiments, consisting in 1000 randomly selected clock cycles, are shown in Table 2. In all cases, the standard deviation of the delays between the compared sampling clocks is below twice the period of the reconstruction clock, which is 12.5 ns.

**Figure 4:**
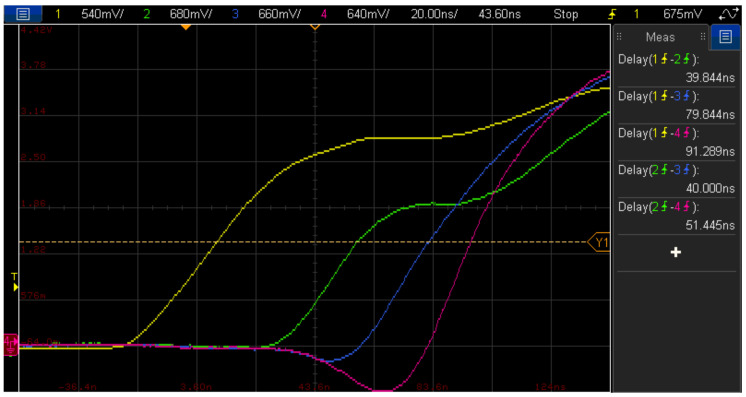
Global sync clock (yellow) and sampling clocks on the accelerometer side with 1 m (green), 5 m (blue) and 10 m (red) cable.

It is recommended that all the ethernet cables connecting the Back-end units to the accelerometers have the same length. If this is not the case, the signal propagation may cause different delays in the clock signal arriving at each accelerometer. To analyse the effect of this delay, the acquisition clock signals at the end of the accelerometer connection cable have been compared using different cable lengths (1, 5 and 10 m). The obtained results, shown in Figure 4 with an oscilloscope screenshot similar to the one presented in Figure 3, evidence that as the cable length increases, the delay introduced in the sampling clock also grows.

The measured delay values shown in Table 3 help to verify that, including with the longest cable length, the delay between the synchronization clocks is no greater than 55 ns and its deviation remains below 12.5 ns, as before. Considering that the maximum sampling period is 250 µs, due to the sampling frequency being 4 kHz, it can be concluded that the data acquired using the distributed system is synchronous enough for the purposes intended in this work, which is the experimental modal identification of a structure, as explained in the next section.

## 4. Validation of the Proposed Distributed System: Modal Analysis

### 4.1. Measurement Layout and FRF Estimation for Modal Analysis

**Figure 5:**
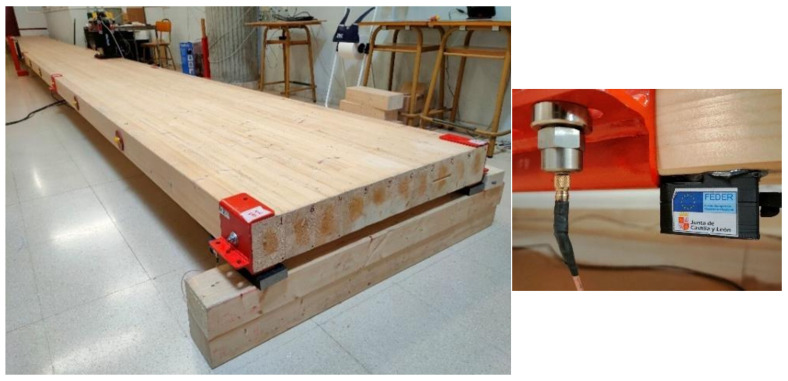
Picture of the structure under test (**left**) and pair of collocated sensors (**right**).

In order to validate the distributed system as a tool to estimate or identify the modal properties of a structure by comparing the results it provides with the ones issued by the reference system, both are installed on the structure depicted in Figure 5. It is a timber platform composed of 10 tightly attached timber beams with a total length of 13.5 m, a height of 140 mm and a width of 100 mm each. The beams are placed side-by-side, which confers the platform the same heigh of 140 mm and a total width of 1 m. The material of the 10 beams is GLULAM 24 h. The platform is simply supported at its both ends, and two sets of three springs (with an elastic constant of approximately 6600 N/m each) are located at the middle section (one per side) to compensate for the self-weight deflection.

**Figure 6:**
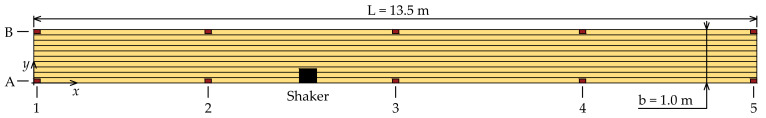
Top view of the measurement layout for the validation tests.

Ten accelerometers of each type are placed on the structure drawing a 5 × 2 grid, as shown in Figure 6; thus, each grid point has a pair of accelerometers: one piezoelectric sensor and one MEMS sensor. To name the grid points, longitudinal sections are evenly separated, named with numbers from 1 to 5, while letters A and B indicate the side of the structure to which the point belongs (side A corresponds to *y* = 0 m and side B, to *y* = 1 m). In addition, to induce a controlled force on the structure, an electromechanical shaker is placed on it, as shown in Figure 6. It is placed on a point of the structure between Section 2 and Section 3, which is not included in the 5 × 2 grid; thus, it excites as many modes as possible in the frequency range of interest. Due to this, two more accelerometers of each type are required: one pair is placed on its moving mass and another pair is located on its frame (the part rigidly attached to the structure). The force induced by the shaker on the structure can be estimated by multiplying the measured acceleration of the moving mass by the mass value, which is 31.2 kg in this case. Although the second pair of accelerometers, placed on the frame of the shaker, does not belong to the 5 × 2 grid, it is of vital importance to extract and scale the mode shapes since they constitute the known as driving point.

**Figure 7:**
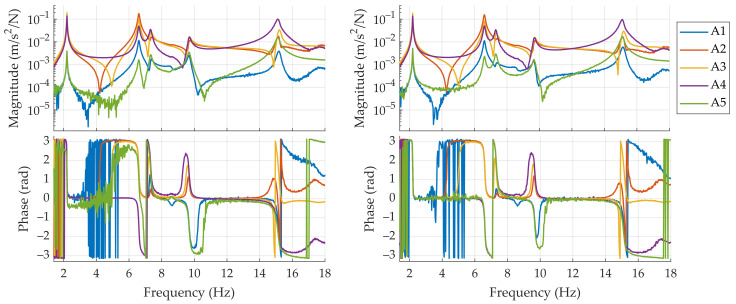
FRFs corresponding to points A1 to A5 estimated by means of the reference system (**left**) and the proposed system (**right**).

As mentioned in Section 2.1, the shaker is controlled by one Back-end unit of the proposed low-cost system, which generates a random noise with frequency components between 0 and 100 Hz. Both logging systems record the response of the twelve installed pairs of accelerometers at 4000 S/s during 660 s. In order to estimate the frequency response functions, which is done separately for each system by using the recordings of the corresponding set of 12 accelerometers, the estimated force induced by the shaker is used as the input to the system and the remaining 11 accelerometers are considered to be the output of the system, leading to a total of 11 FRFs. To perform the FRFs computation, the time signals are segmented by means of a Hanning window of 120 s length (480,000 samples), leading to a frequency resolution of 0.0167 Hz. Two consecutive segments are overlapped 50% of their length (240,000 samples); thus, a total of 10 averages can be computed after processing the whole record. A custom LabVIEW software is used to simultaneously record and compute the FRFs associated to the proposed system, whilst an external software, provided with the commercial hardware, is used to apply the same procedure to the data recorded with it. The computed FRFs associated to the side A of the structure are shown in Figure 7. Subtle differences can be appreciated between both sets of FRFs, which are mainly due to slight misplacements of the sensors that compose each co-located pair (see Figure 5, right picture). However, these differences are not significant in the surroundings of main peaks of the magnitude plots, which are representative of the structural dynamic behaviour. Due to that, the modal parameter identification performed from them in the next sections is not compromised in any way.

### 4.2. Modal Analysis Procedures

The FRFs estimated by means of the proposed system are processed in order to estimate the properties of a set of modes, namely their natural frequency, damping ratio and mode shape. The identified modal properties are compared to the ones obtained after processing the FRFs issued by the reference system with a more robust and powerful method. In this section, the algorithms used to identify the modal properties are described and the next section is devoted to compare and discuss the obtained results.

#### 4.2.1. Proposed System Identification Method (FDPI)

As mentioned in Section 2, the proposed system is entirely based on the myRIO platform and the LabVIEW environment, and thus is the software module which performs the modal identification. The modal analysis software module implements the Frequency Domain Parameter Identification algorithm (FDPI) [35,36], which belongs to the so-called direct methods since, starting from the calculated FRFs, it directly estimates the submatrices of the state transition matrix of a state space representation of the system such as the one shown in Equation (1), where $q\left(t\right)$ is the n×1 DOF vector, $A_{K}$ and $A_{C}$ are the submatrices of interest of dimension n×n, M is the mass matrix of the structure of dimension n×n, $f\left(t\right)$ is the force vector of dimension n×1 and ∅ accounts for a vector or matrix of the appropriate dimension. Note that the spatial discretization, which determines the elements of vector $q\left(t\right)$, is established according to the number and position of the accelerometers on the structure. After the estimation of the submatrices $A_{K}$ and $A_{C}$, the modal properties can be calculated by solving the second order eigenproblem shown in Equation (2), where $s_{r}$ stands for the complex eigenvalues or poles of the system and $\phi_{r}$ are the complex eigenvectors or mode shapes. The eigenvalues or poles $s_{r}$ are closely related to the natural frequencies $\omega_{r}$ and the damping ratios $\zeta_{r}$ through the expression $s_{r}=-\omega_{r}\zeta_{r}+j\omega_{r}\sqrt{1-\zeta_{r}^{2}}$, where j is the imaginary unit; thus, $j^{2}=-1$.

(1) $$\left[\begin{array}{c} \dot{q}\left(t\right)\\ \ddot{q}\left(t\right) \end{array}\right]=\left[\begin{array}{cc} I & \emptyset \\ -A_{K} & -A_{C} \end{array}\right]\left[\begin{array}{c} q\left(t\right)\\ \dot{q}\left(t\right) \end{array}\right]+\left[\begin{array}{c} \emptyset \\ M^{-1} \end{array}\right]f\left(t\right)$$

(2) $$\left(s_{r}^{2}+A_{C}s_{r}+A_{K}\right)\phi_{r}=0$$

To estimate a model such as the one presented in Equation (1), an overdetermined system of equations is solved in the least-squares sense. The unknowns of the system of equations are the elements of matrices $A_{C}$ and $A_{K}$, which are related to the calculated FRFs, $H\left(\omega \right)$, through the expression in Equation (3), where $I_{n}$ is the n×n identity matrix. Note that this expression is verified for every calculated frequency line ω inside the frequency range of interest; thus, the number of equations in the system linearly increases with the number of calculated lines. Finally, extra terms can be added to the right hand of Equation (3) to account for the influence of modes outside the measured frequency range, as explained in [37].

(3) $$\left(-\omega^{2}I_{n}+j\omega A_{C}+A_{K}\right)H\left(\omega \right)=M^{-1}$$

As stated before, a specific implementation of this modal identification method is programmed in LabVIEW; thus, it can be easily integrated in the software used to control the proposed system. It is suitable to identify modes with low to moderate damping level. However, it does not handle well with wide frequency ranges containing a high number of modes; thus, large frequency bands must be segmented before applying this identification method. For that reason, in order to obtain the results obtained in Section 4.3, the frequency range is segmented in sections containing up to three peaks each and the algorithm is applied on each one.

#### 4.2.2. Reference System Identification Method (CF)

Conversely, the reference system is completed by a custom MATLAB software package intended to identify the modal properties of a certain set of modes from some computed FRFs. The algorithm consists in performing a curve-fitting of the FRFs to the linear expression shown in Equation (4), which corresponds to a single FRF between the degree of freedoms *i* and *k* [38]. In that expression, the synthesized FRF is obtained by means of the shown modal properties, where $\phi_{ri}$ corresponds to the i-th component of the r-th mode shape and $s_{r}$ is the eigenvalue or pole, closely related to the natural frequency $\omega_{r}$ and the damping ratio $\zeta_{r}$ as stated before, and the asterisk symbol * stands for the complex conjugate.

(4) $$h_{ik}\left(\omega \right)=\overset{n}{\underset{r=1}{\sum }}\left(\frac{\phi_{ri}\phi_{rk}}{j\omega -s_{r}}+\frac{\phi_{ri}^{*}\phi_{rk}^{*}}{j\omega -s_{r}^{*}}\right)$$

An optimization procedure is carried out to obtain the optimal values of the modal parameters which minimize the error between the computed and the synthesized FRFs. The cost function J which is minimized during the optimization procedure is shown in Equation (5), where $Re\left(\cdot \right)$ and $Im\left(\cdot \right)$ stand, respectively, for the real and imaginary parts, and the tilde (~) represents the experimental FRF. The sum is extended to the n calculated FRFs, assuming i standing for the reference DOF (where the force is applied) and k the response DOFs. As can be seen in Equation (5), the optimization procedure considers both the real and the imaginary parts, instead of the magnitude and phase of the FRFs. This is due to the fact that the available algorithms that compute the phase component return values between −π and π rad (thus leading to vertical lines in the phase plots, as shown in Figure 7) and computing the difference between values at both sides of each bound may lead to unrealistic high error values.

(5) $$J=\overset{n}{\underset{k=1}{\sum }}\left\{{\left(Re\left(h_{ik}\right)-Re\left({\tilde{h}}_{ik}\right)\right)}^{2}+{\left(Im\left(h_{ik}\right)-Im\left({\tilde{h}}_{ik}\right)\right)}^{2}\right\}$$

The whole optimization procedure is repeated several times assuming different number of modes, n. First, the lowest value, $n_{0}$, is computed with randomly generated initial guesses for the modal properties. The optimal values for that scenario are used as the initial guess for the next one, $n_{1}=n_{0}+1$, together with the set of modal properties associated to the new considered mode, which are again randomly generated. The process is sequentially repeated until the value of the cost function, J, is low enough or a certain value for n is reached. Due to the randomness of the initial guesses, each scenario is repeated several times and the best fit is kept as the optimal solution for that stage.

The proposed method is potentially long to be applied and requires much computational resources. For this reason, it is not suitable to be implemented in a low-cost environment and has been programmed and run in a powerful PC by making use of MATLAB functions such as lsqnonlin(). Conversely, it is a robust methodology capable of identifying a high number of modes in a wide frequency band with a damping level from low to moderate; thus, it is a good way to obtain trustfully estimates to compare with.

### 4.3. Results Comparison

By means of the previously computed FRFs, the modal properties (natural frequencies, damping ratios and mode shapes) of the platform are obtained by applying one of the procedures explained in Section 4.2. The identified natural frequencies and damping ratios are shown in Table 4. The values obtained with the proposed system and the FDPI algorithm are compared to the ones obtained with the reference system and the CF algorithm by means of the relative error, whose expression is shown in Equation (6) and where the symbol x may stand for the natural frequency or the damping ratio. As can be seen, in order to compute this relative error, the estimates provided by the reference system (CF) are taken as the trustfully ones and the properties obtained by means of the proposed system (FDPI) are compared against them.

(6) $$\epsilon =100\cdot \frac{x_{\mathrm{FDPI}}-x_{\mathrm{CF}}}{x_{\mathrm{CF}}}$$

The mode shapes are compared through the known as Modal Assurance Criterion (MAC) [38], whose mathematical expression is shown in Equation (7), which is a coefficient conceived to compare two complex column vectors $\phi_{i}$ and $\phi_{k}$, regardless their scaling and rotation in the complex plane. If both compared complex vectors are equivalent or similar, except for their scaling and rotation, the computed MAC value equals 1. Otherwise, the value decreases towards 0, when both vectors can be said to be completely different.

(7) $$MAC_{ik}=\frac{{\left|\phi_{i}^{*t}\phi_{k}\right|}^{2}}{\left(\phi_{i}^{*t}\phi_{i}\right)\left(\phi_{k}^{*t}\phi_{k}\right)}$$

As can be seen in Table 4, there is a high correspondence between the modal properties estimated by means of the proposed system and the ones estimated by using the reference system. The relative error between both sets of natural frequencies, is under 1.1% in all cases, being the difference smaller than the frequency resolution (0.0167 Hz) in some of them. Conversely, the damping ratios show higher error values, greater than 15% in two cases. However, due to its nature, this magnitude is always affected by higher uncertainty levels, and it is usual for it to evidence more variability than the natural frequencies. In this sense, processing the same FRFs by using different algorithms can provide significantly different estimates of the damping ratios; thus, the results obtained here are considered to be within the normal bounds.

**Figure 8:**
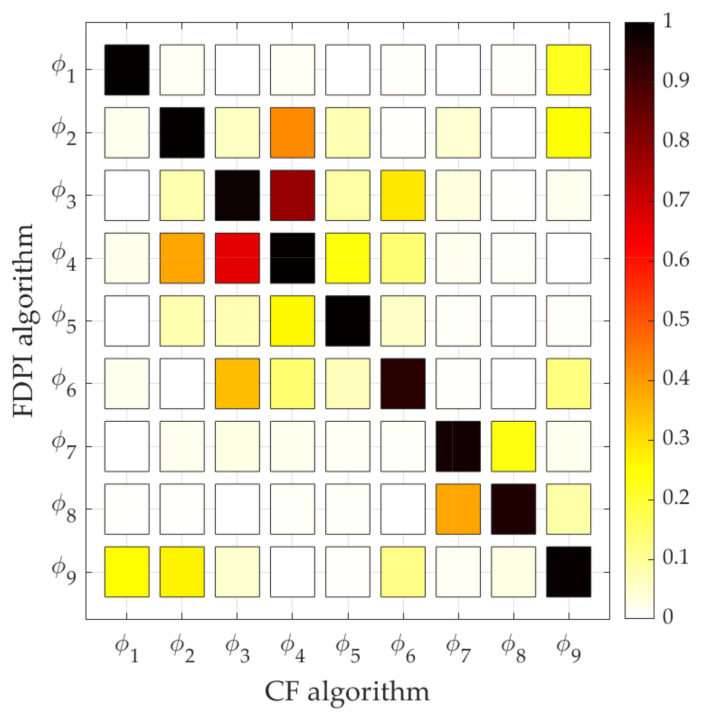
MAC matrix computed by comparing the mode shapes estimated through the CF algorithm (horizontal axis) and the FDPI algorithm (vertical axis).

Finally, the MAC column of Table 4 shows the success in estimating the mode shapes. As mentioned before, this value increases towards 1 when two modes shapes are similar. This is the case for all modes, but specially for modes 1, 2, 4 and 5, whose MAC value is greater than 0.99. Apart from calculating and analysing the MAC of corresponding mode shapes (same mode number), it is also interesting to make a more complete comparison by calculating the MAC value associated to the mode shapes of different mode number. It is expected for those MAC values to be significantly lower to 1 if the mode shapes are different enough. The complete comparison is made in Figure 8, which shows he known as MAC matrix, where the mode shapes associated to the algorithm CF are placed on the horizontal axis and the ones associated to the algorithm FDPI are placed on the vertical axis. The white colour corresponds to a null MAC value, whereas the solid black colour indicates a MAC value that equals 1. Intermediate values are represented with yellow, orange and red colours in ascending order. As can be seen, the main diagonal is mostly black, according to the values in the last column of Table 4, whereas the values outside the main diagonal are mostly yellow and white, evidencing the lack of similarities between the rest of pairs of mode shapes, as expected.

**Figure 9:**
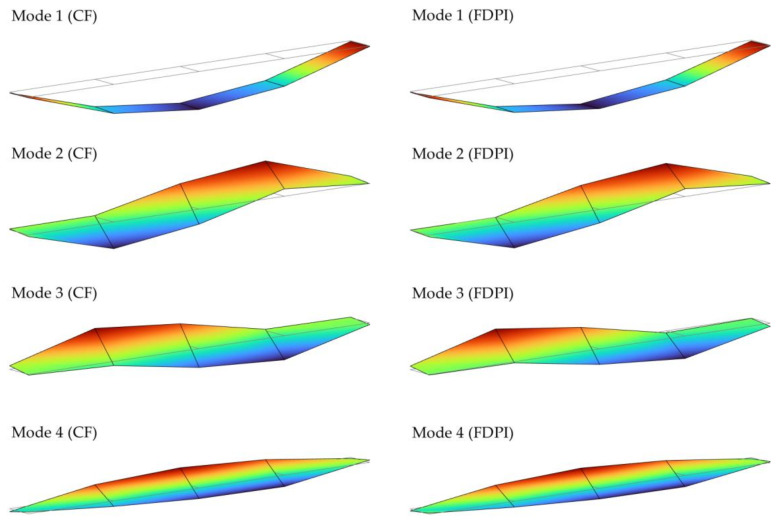
Mode shapes corresponding to modes 1 to 4 estimated by applying both algorithms: CF (**left**) and FDPI (**right**).

This can also be seen when plotting the mode shapes. Figure 9 shows, as an example, four selected mode shapes extracted with both algorithms. As was anticipated by the MAC values shown in Figure 8, the mode shapes associated to modes 1, 2 and 3 are different to each other because the out-of-diagonal terms are less than 0.1. Although they are somehow similar, the modes shapes of modes 2 and 3 have vibration nodes (null mode shape coordinates) at different locations, making them completely different in terms of MAC comparison. Conversely, mode shapes of modes 3 and 4 are more similar, as the MAC values between 0.6 and 0.8 reveal, since the modal coordinates outlines a similar shape in many points. Finally, note that this comparison can only be made according to the modal coordinates associated to the measured points and that intermediate points (linearly interpolated in Figure 9) do not take part in any way.

## 5. Conclusions

In this work, the scalability of a low-cost monitoring system has been tested in a real scenario. Several units based on a myRIO platform together with some digital MEMS accelerometers have been connected in order to synchronously acquire data from all sensors. As shown, the ability to synchronize all the devices has been validated as well as its applicability to perform the complete modal analysis of a structure.

The most relevant remarks can be synthesised as follows:

- The low-cost system consisting of three myRIOs and twelve MEMS accelerometers has been installed on a structure in parallel to other more sophisticated reference system, commercially available for modal analysis purposes.
- After recording the time domain signals and calculating the associate Frequency Response Functions, the modal parameters of the structure have been estimated by different means: a robust but slow and computationally resource-intensive algorithm has been used to process the reference data within the MATLAB environment, whilst a simpler algorithm, implemented in the LabVIEW environment, has been used to process the low-cost system data.
- Due to the high synchronization attained by means of the proposed system, the modal properties estimated with it are similar to the ones estimated by using the commercial hardware and software, with relative errors under 1.1% for the natural frequencies and under 17% for the damping ratios.
- The mode shapes have been compared via the Modal Assurance Criterion, obtaining values above 0.95 in all cases.

With this, it can be easily concluded that the proposed low-cost system is able to synchronously measure data useful to accurately determine the modal properties of a structure.

## Figures and Tables

**Table 1:** Main characteristics of the proposed and reference system.

Characteristic	Proposed System	Reference System
Accel. range	±2 g, ±4 g, and ±8 g	±60 g
Accel. digital sensitivity	3.9, 7.8 and 15.6 μg/LSB	11.9 μg/LSB
Accel. noise density	25 µg/√Hz	3 µg/√Hz
Max. sample frequency	4 kHz	200 kHz
Bits per sample	20	24
Max. accelerometer channels	6 tri-axial per device	16 uni-axial
Total cost (device + 6 accels)	€928 per device	€9050 per device

**Table 2:** Measured delays between sync and sampling clocks.

	Delay (ns)			
	Mean	Min	Max	Std. Dev.
Sync clock-Master unit	48.92	45.54	62.73	1.13
Sync clock-Slave unit 1	51.36	46.54	63.44	1.57
Sync clock-Slave unit 2	54.39	49.22	65.70	1.40
Master unit-Slave unit 1	2.43	−4.53	7.81	2.11
Master unit-Slave unit 2	5.46	−1.47	9.92	1.96

**Table 3:** Clock delays between sync clock and sampling clocks including propagation delays.

	Delay (ns)			
	Mean	Min	Max	Std. Dev
Sync clock-Master unit	40.04	38.75	57.27	1.47
Sync clock-Slave unit 1	79.67	74.92	83.78	1.50
Sync clock-Slave unit 2	90.74	87.77	93.44	1.00
Master unit-Slave unit 1	39.62	35.23	43.60	2.25
Master unit-Slave unit 2	50.703	41.69	53.16	1.72

**Table 4:** Estimated modal properties.

Mode	Natural Frequency (Hz)			Damping Ratio (%)			MAC
	CF	FDPI	Error (%)	CF	FDPI	Error (%)	
1	2.198	2.190	−0.377	0.389	0.436	11.9	0.999
2	6.602	6.600	−0.0492	0.709	0.744	4.91	0.997
3	7.324	7.361	0.508	0.910	1.05	15.4	0.981
4	9.685	9.669	−0.165	0.802	0.812	1.35	0.995
5	15.07	15.05	−0.135	0.931	0.779	−16.3	0.994
6	24.15	24.14	−0.0374	0.684	0.674	−1.55	0.942
7	26.79	26.71	−0.324	1.10	1.08	−2.10	0.973
8	28.23	28.06	−0.599	1.08	1.20	10.6	0.953
9	39.56	39.13	−1.08	0.977	1.02	4.80	0.989

## Data Availability

The validation data used to support the findings of this study are available from the corresponding author upon request.
